# Di-μ-acetato-κ^4^ *O*:*O*-bis­({2-[(piperidin-2-ylmeth­yl)imino­meth­yl]phenolato-κ^3^ *N*,*N*′,*O*}copper(II)) monohydrate

**DOI:** 10.1107/S1600536812023070

**Published:** 2012-05-26

**Authors:** Xiao-qin Wang

**Affiliations:** a Department of Medicinal Chemistry and Pharm-analysis, Guangdong Medical College, Dong guan, People’s Republic of China

## Abstract

In the binuclear centrosymmetric title compound, [Cu_2_(C_13_H_17_N_2_O)_2_(C_2_H_3_O_2_)_2_]·H_2_O, the Cu^II^ atom is coordin­ated by two N atoms and one O atom from the Schiff base ligand and an acetate O atom in a distorted suare-planar geometry. The water O atom is invoved in three different hydrogen-bonding interactions, as donor to the acetate O atom and to the the ligand O atom and as acceptor to a ligand N atom.

## Related literature

The ligand was prepared according to a literature method, see: Greatti *et al.* (2008 ▶).
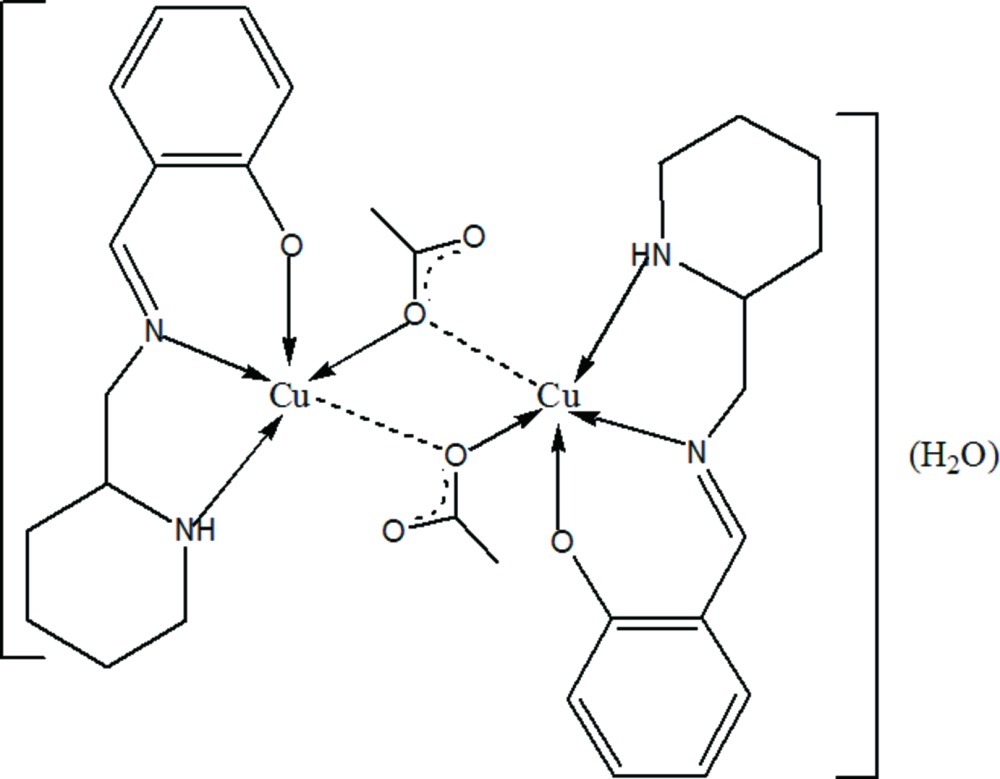


## Experimental

### 

#### Crystal data

[Cu_2_(C_13_H_17_N_2_O)_2_(C_2_H_3_O_2_)_2_]·H_2_O
*M* *_r_* = 715.79
Triclinic, 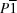

*a* = 8.7725 (18) Å
*b* = 8.8259 (18) Å
*c* = 11.894 (2) Å
α = 101.98 (3)°
β = 101.04 (3)°
γ = 110.13 (3)°
*V* = 810.4 (3) Å^3^
*Z* = 1
Mo *K*α radiation
μ = 1.37 mm^−1^
*T* = 292 K
0.20 × 0.10 × 0.10 mm

#### Data collection

Bruker APEXII diffractometer
Absorption correction: multi-scan (*SADABS*; Sheldrick, 1996 ▶) *T*_min_ = 0.772, *T*_max_ = 0.876
7559 measured reflections
3542 independent reflections
2473 reflections with *I* > 2σ(*I*)
*R*_int_ = 0.034

#### Refinement

*R*[*F* ^2^ > 2σ(*F* ^2^)] = 0.041
*wR*(*F* ^2^) = 0.136
*S* = 1.24
3542 reflections
211 parameters
H atoms treated by a mixture of independent and constrained refinement
Δρ_max_ = 0.55 e Å^−3^
Δρ_min_ = −0.84 e Å^−3^

### 

Data collection: *APEX2* (Bruker, 2005 ▶); cell refinement: *SAINT* (Bruker, 2005 ▶); data reduction: *SAINT*; program(s) used to solve structure: *SHELXS97* (Sheldrick, 2008 ▶); program(s) used to refine structure: *SHELXL97* (Sheldrick, 2008 ▶); molecular graphics: *X-SEED* (Barbour, 2001 ▶); software used to prepare material for publication: *publCIF* (Westrip, 2010) ▶.

## Supplementary Material

Crystal structure: contains datablock(s) I, global. DOI: 10.1107/S1600536812023070/gw2117sup1.cif

Structure factors: contains datablock(s) I. DOI: 10.1107/S1600536812023070/gw2117Isup2.hkl

Additional supplementary materials:  crystallographic information; 3D view; checkCIF report

## Figures and Tables

**Table 1:** Hydrogen-bond geometry (Å, °)

*D*—H⋯*A*	*D*—H	H⋯*A*	*D*⋯*A*	*D*—H⋯*A*
O1*W*—H1*W*2⋯O2^i^	0.79 (6)	2.06 (6)	2.845 (5)	173 (6)
O1*W*—H1*W*1⋯O3^ii^	0.81 (8)	2.24 (9)	2.970 (6)	151 (8)
N2—H1*N*⋯O1*W*^iii^	1.00 (5)	2.09 (5)	3.047 (5)	159 (4)

Symmetry codes: (i) 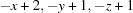; (ii) 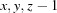; (iii) 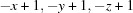.

**Fig. 1.**
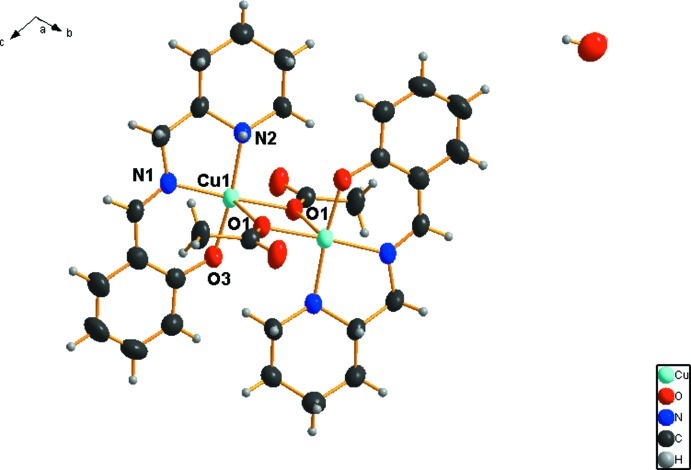
Thermal ellipsoid plot (Barbour, 2001) of [Cu2(C13H17N2O)2(C2H3O2)2](H2O), at the 30% probability level; hydrogen atoms are drawn as spheres of arbitrary radius.

## supplementary crystallographic information

### Comment

There are no crystal structure studies of metal complexes of the new tridentate Schiff ligand. In the title compound, the binuclear molecule is centrosymmetric and the copper atom adopts a distorted square geometry, coordinated by N1, N2, O3 from the ligand and O1 from acetate. There are three kinds of hydrogen bonging in O1w of the lattice water with O2 from acetate, O3 and N2 from ligand. Related hydrogen bonding distances are listed in Table 1.

### Experimental

0.12 g (1 mmol) of salicylaldehyde and 0.12 g (1 mmol) of 2-(aminomethyl)piperidine were dissolved in 10 ml of methanol. The solution was stirred at room temperature for 1 h and 0.20 g (1 mmol) monohydrate copper(II) acetate was added. The reaction was stirred at room temperature for 30 minutes. The crude product was collected by filtration and then washed with methanol. Blue block shaped crystals suitable for single-crystal X-ray study were obtained by recrystallization from 2:1 MeCN-MeOH solution (5 ml) with the yield of 66%.CH&N elemental analysis. Found (calcd): C, 50.59 (50.29); H, 6.18 (6.15); N, 8.02 (7.82).

### Refinement

Carbon-bound H-atoms were placed in calculated positions (C—H 0.95 to 0.99 Å) and were included in the refinement in the riding model approximation, with *U*(H) set to 1.2 to 1.5*U*(C).

The amino H-atom was located in a difference Fourier map, and was refined with a distance restraint of N–H 0.88±0.01 Å; its temperature factor was freely refined.

The final difference Fourier map had a peak in the vicinity of Zn1 but was otherwise featureless.

### Crystal data

**Table:**

[Cu_2_(C_13_H_17_N_2_O)_2_(C_2_H_3_O_2_)_2_]·H_2_O	*Z* = 1
*M**_r_* = 715.79	*F*(000) = 374
Triclinic, *P*1	*D*_x_ = 1.467 Mg m^−^^3^
*a* = 8.7725 (18) Å	Mo *K*α radiation, λ = 0.71073 Å
*b* = 8.8259 (18) Å	Cell parameters from 7559 reflections
*c* = 11.894 (2) Å	θ = 3.4–27.5°
α = 101.98 (3)°	µ = 1.37 mm^−^^1^
β = 101.04 (3)°	*T* = 292 K
γ = 110.13 (3)°	Block, blue
*V* = 810.4 (3) Å^3^	0.20 × 0.10 × 0.10 mm

### Data collection

**Table:**

Bruker APEXII diffractometer	3542 independent reflections
Radiation source: fine-focus sealed tube	2473 reflections with *I* > 2σ(*I*)
Graphite monochromator	*R*_int_ = 0.034
ω scans	θ_max_ = 27.5°, θ_min_ = 3.4°
Absorption correction: multi-scan (*SADABS*; Sheldrick, 1996)	*h* = −11→11
*T*_min_ = 0.772, *T*_max_ = 0.876	*k* = −10→11
7559 measured reflections	*l* = −15→15

### Refinement

**Table:**

Refinement on *F*^2^	Primary atom site location: structure-invariant direct methods
Least-squares matrix: full	Secondary atom site location: difference Fourier map
*R*[*F*^2^ > 2σ(*F*^2^)] = 0.041	Hydrogen site location: inferred from neighbouring sites
*wR*(*F*^2^) = 0.136	H atoms treated by a mixture of independent and constrained refinement
*S* = 1.24	*w* = 1/[σ^2^(*F*_o_^2^) + (0.0652*P*)^2^] where *P* = (*F*_o_^2^ + 2*F*_c_^2^)/3
3542 reflections	(Δ/σ)_max_ = 0.001
211 parameters	Δρ_max_ = 0.55 e Å^−^^3^
0 restraints	Δρ_min_ = −0.84 e Å^−^^3^

### Special details

**Table:**

Geometry. All e.s.d.'s (except the e.s.d. in the dihedral angle between two l.s. planes) are estimated using the full covariance matrix. The cell e.s.d.'s are taken into account individually in the estimation of e.s.d.'s in distances, angles and torsion angles; correlations between e.s.d.'s in cell parameters are only used when they are defined by crystal symmetry. An approximate (isotropic) treatment of cell e.s.d.'s is used for estimating e.s.d.'s involving l.s. planes.

### Fractional atomic coordinates and isotropic or equivalent isotropic displacement parameters (Å^2^)

**Table:**

	*x*	*y*	*z*	*U*_iso_*/*U*_eq_	
Cu1	0.49939 (5)	0.30738 (5)	1.01386 (3)	0.03597 (17)	
O1	0.6331 (3)	0.4839 (3)	0.9559 (2)	0.0377 (5)	
O2	0.7691 (4)	0.3218 (3)	0.9094 (3)	0.0531 (7)	
O3	0.6639 (3)	0.4046 (3)	1.1697 (2)	0.0440 (6)	
N1	0.3578 (4)	0.1242 (4)	1.0631 (2)	0.0376 (6)	
N2	0.3251 (4)	0.1769 (4)	0.8508 (2)	0.0401 (7)	
C1	0.7429 (5)	0.4504 (4)	0.9107 (3)	0.0381 (7)	
C2	0.8387 (6)	0.5763 (5)	0.8546 (4)	0.0541 (10)	
H2A	0.9169	0.5389	0.8231	0.081*	
H2B	0.9000	0.6844	0.9141	0.081*	
H2C	0.7604	0.5858	0.7908	0.081*	
C3	0.6383 (5)	0.3757 (4)	1.2699 (3)	0.0411 (8)	
C4	0.7613 (6)	0.4822 (5)	1.3792 (3)	0.0554 (10)	
H4A	0.8585	0.5680	1.3781	0.066*	
C5	0.7393 (7)	0.4609 (6)	1.4868 (4)	0.0724 (14)	
H5A	0.8207	0.5344	1.5577	0.087*	
C6	0.5978 (7)	0.3316 (7)	1.4920 (4)	0.0782 (15)	
H6A	0.5833	0.3192	1.5655	0.094*	
C7	0.4810 (7)	0.2237 (6)	1.3877 (3)	0.0645 (12)	
H7A	0.3879	0.1348	1.3907	0.077*	
C8	0.4966 (5)	0.2425 (5)	1.2753 (3)	0.0457 (9)	
C9	0.3696 (5)	0.1199 (4)	1.1711 (3)	0.0407 (8)	
H9A	0.2882	0.0287	1.1827	0.049*	
C10	0.2178 (5)	−0.0093 (5)	0.9659 (3)	0.0476 (9)	
H10A	0.1134	0.0057	0.9662	0.057*	
H10B	0.2041	−0.1185	0.9770	0.057*	
C11	0.2541 (5)	−0.0029 (4)	0.8480 (3)	0.0444 (8)	
H11A	0.3430	−0.0453	0.8431	0.053*	
C12	0.1037 (6)	−0.1121 (5)	0.7406 (3)	0.0539 (10)	
H12A	0.0094	−0.0806	0.7468	0.065*	
H12B	0.0700	−0.2294	0.7394	0.065*	
C13	0.1453 (6)	−0.0928 (5)	0.6245 (3)	0.0600 (11)	
H13A	0.2260	−0.1421	0.6114	0.072*	
H13B	0.0431	−0.1531	0.5578	0.072*	
C14	0.2180 (6)	0.0888 (5)	0.6292 (3)	0.0543 (10)	
H14A	0.1303	0.1322	0.6285	0.065*	
H14B	0.2547	0.0978	0.5580	0.065*	
C15	0.3660 (5)	0.1960 (5)	0.7388 (3)	0.0473 (9)	
H15A	0.4605	0.1645	0.7332	0.057*	
H15B	0.4007	0.3136	0.7409	0.057*	
O1W	0.9643 (5)	0.7056 (5)	0.1856 (4)	0.0694 (10)	
H1N	0.230 (7)	0.211 (6)	0.859 (4)	0.085 (16)*	
H1W1	0.908 (11)	0.625 (10)	0.203 (7)	0.16 (4)*	
H1W2	1.033 (8)	0.696 (8)	0.153 (5)	0.10 (2)*	

### Atomic displacement parameters (Å^2^)

**Table:**

	*U*^11^	*U*^22^	*U*^33^	*U*^12^	*U*^13^	*U*^23^
Cu1	0.0363 (3)	0.0324 (2)	0.0375 (2)	0.00788 (19)	0.01277 (17)	0.01509 (17)
O1	0.0356 (14)	0.0359 (12)	0.0444 (13)	0.0106 (11)	0.0183 (11)	0.0178 (10)
O2	0.0530 (18)	0.0478 (15)	0.0771 (19)	0.0241 (15)	0.0353 (15)	0.0341 (14)
O3	0.0374 (15)	0.0459 (14)	0.0419 (13)	0.0051 (12)	0.0094 (11)	0.0211 (11)
N1	0.0361 (17)	0.0389 (15)	0.0404 (15)	0.0120 (14)	0.0135 (12)	0.0195 (12)
N2	0.0419 (19)	0.0388 (16)	0.0378 (14)	0.0091 (15)	0.0149 (13)	0.0163 (13)
C1	0.032 (2)	0.0364 (18)	0.0431 (17)	0.0094 (16)	0.0105 (15)	0.0143 (15)
C2	0.059 (3)	0.050 (2)	0.069 (2)	0.021 (2)	0.039 (2)	0.032 (2)
C3	0.049 (2)	0.0379 (18)	0.0377 (17)	0.0190 (18)	0.0101 (16)	0.0129 (15)
C4	0.064 (3)	0.043 (2)	0.049 (2)	0.013 (2)	0.010 (2)	0.0138 (18)
C5	0.092 (4)	0.068 (3)	0.037 (2)	0.021 (3)	0.007 (2)	0.005 (2)
C6	0.093 (4)	0.089 (4)	0.038 (2)	0.016 (3)	0.023 (2)	0.021 (2)
C7	0.072 (3)	0.073 (3)	0.045 (2)	0.018 (3)	0.026 (2)	0.023 (2)
C8	0.050 (2)	0.051 (2)	0.0424 (18)	0.023 (2)	0.0174 (17)	0.0175 (17)
C9	0.039 (2)	0.0374 (18)	0.0494 (19)	0.0115 (17)	0.0174 (16)	0.0215 (16)
C10	0.039 (2)	0.0402 (19)	0.050 (2)	−0.0002 (17)	0.0073 (16)	0.0192 (16)
C11	0.043 (2)	0.0354 (18)	0.0478 (19)	0.0063 (17)	0.0102 (16)	0.0166 (15)
C12	0.050 (3)	0.043 (2)	0.052 (2)	0.0030 (19)	0.0039 (18)	0.0180 (18)
C13	0.062 (3)	0.054 (2)	0.048 (2)	0.011 (2)	0.006 (2)	0.0130 (19)
C14	0.063 (3)	0.053 (2)	0.0387 (18)	0.014 (2)	0.0096 (18)	0.0171 (17)
C15	0.053 (3)	0.047 (2)	0.0376 (17)	0.0107 (19)	0.0141 (17)	0.0188 (16)
O1W	0.061 (2)	0.064 (2)	0.091 (3)	0.032 (2)	0.030 (2)	0.0183 (19)

### Geometric parameters (Å, º)

**Table:**

Cu1—O3	1.928 (3)	C6—H6A	0.9300
Cu1—O1	1.944 (2)	C7—C8	1.408 (5)
Cu1—N1	1.946 (3)	C7—H7A	0.9300
Cu1—N2	2.037 (3)	C8—C9	1.426 (5)
O1—C1	1.277 (4)	C9—H9A	0.9300
O2—C1	1.230 (4)	C10—C11	1.504 (5)
O3—C3	1.313 (4)	C10—H10A	0.9700
N1—C9	1.278 (4)	C10—H10B	0.9700
N1—C10	1.459 (5)	C11—C12	1.506 (5)
N2—C15	1.472 (4)	C11—H11A	0.9800
N2—C11	1.482 (4)	C12—C13	1.522 (6)
N2—H1N	1.00 (5)	C12—H12A	0.9700
C1—C2	1.505 (5)	C12—H12B	0.9700
C2—H2A	0.9600	C13—C14	1.491 (6)
C2—H2B	0.9600	C13—H13A	0.9700
C2—H2C	0.9600	C13—H13B	0.9700
C3—C8	1.410 (6)	C14—C15	1.508 (5)
C3—C4	1.412 (5)	C14—H14A	0.9700
C4—C5	1.372 (6)	C14—H14B	0.9700
C4—H4A	0.9300	C15—H15A	0.9700
C5—C6	1.390 (7)	C15—H15B	0.9700
C5—H5A	0.9300	O1W—H1W1	0.81 (8)
C6—C7	1.359 (6)	O1W—H1W2	0.79 (6)
			
O3—Cu1—O1	91.10 (11)	C7—C8—C9	117.6 (4)
O3—Cu1—N1	91.94 (12)	C3—C8—C9	123.0 (3)
O1—Cu1—N1	176.92 (10)	N1—C9—C8	125.7 (4)
O3—Cu1—N2	173.00 (11)	N1—C9—H9A	117.1
O1—Cu1—N2	93.92 (11)	C8—C9—H9A	117.1
N1—Cu1—N2	83.01 (12)	N1—C10—C11	109.4 (3)
C1—O1—Cu1	114.3 (2)	N1—C10—H10A	109.8
C3—O3—Cu1	126.2 (2)	C11—C10—H10A	109.8
C9—N1—C10	119.3 (3)	N1—C10—H10B	109.8
C9—N1—Cu1	125.8 (3)	C11—C10—H10B	109.8
C10—N1—Cu1	114.6 (2)	H10A—C10—H10B	108.2
C15—N2—C11	111.7 (3)	N2—C11—C10	107.8 (3)
C15—N2—Cu1	121.4 (3)	N2—C11—C12	113.2 (3)
C11—N2—Cu1	106.9 (2)	C10—C11—C12	113.6 (3)
C15—N2—H1N	110 (3)	N2—C11—H11A	107.3
C11—N2—H1N	102 (3)	C10—C11—H11A	107.3
Cu1—N2—H1N	102 (3)	C12—C11—H11A	107.3
O2—C1—O1	123.3 (3)	C11—C12—C13	111.1 (4)
O2—C1—C2	120.4 (3)	C11—C12—H12A	109.4
O1—C1—C2	116.2 (3)	C13—C12—H12A	109.4
C1—C2—H2A	109.5	C11—C12—H12B	109.4
C1—C2—H2B	109.5	C13—C12—H12B	109.4
H2A—C2—H2B	109.5	H12A—C12—H12B	108.0
C1—C2—H2C	109.5	C14—C13—C12	111.0 (3)
H2A—C2—H2C	109.5	C14—C13—H13A	109.4
H2B—C2—H2C	109.5	C12—C13—H13A	109.4
O3—C3—C8	124.0 (3)	C14—C13—H13B	109.4
O3—C3—C4	118.4 (4)	C12—C13—H13B	109.4
C8—C3—C4	117.6 (3)	H13A—C13—H13B	108.0
C5—C4—C3	121.0 (4)	C13—C14—C15	112.9 (3)
C5—C4—H4A	119.5	C13—C14—H14A	109.0
C3—C4—H4A	119.5	C15—C14—H14A	109.0
C4—C5—C6	121.3 (4)	C13—C14—H14B	109.0
C4—C5—H5A	119.4	C15—C14—H14B	109.0
C6—C5—H5A	119.4	H14A—C14—H14B	107.8
C7—C6—C5	118.7 (4)	N2—C15—C14	112.4 (3)
C7—C6—H6A	120.6	N2—C15—H15A	109.1
C5—C6—H6A	120.6	C14—C15—H15A	109.1
C6—C7—C8	122.0 (5)	N2—C15—H15B	109.1
C6—C7—H7A	119.0	C14—C15—H15B	109.1
C8—C7—H7A	119.0	H15A—C15—H15B	107.9
C7—C8—C3	119.4 (4)	H1W1—O1W—H1W2	118 (6)
			
O3—Cu1—O1—C1	89.0 (2)	C4—C3—C8—C7	1.6 (5)
N2—Cu1—O1—C1	−86.1 (2)	O3—C3—C8—C9	3.1 (6)
O1—Cu1—O3—C3	161.2 (3)	C4—C3—C8—C9	−175.8 (3)
N1—Cu1—O3—C3	−19.3 (3)	C10—N1—C9—C8	−178.6 (3)
O3—Cu1—N1—C9	15.0 (3)	Cu1—N1—C9—C8	−5.0 (5)
N2—Cu1—N1—C9	−169.8 (3)	C7—C8—C9—N1	174.9 (4)
O3—Cu1—N1—C10	−171.1 (3)	C3—C8—C9—N1	−7.7 (6)
N2—Cu1—N1—C10	4.1 (2)	C9—N1—C10—C11	−165.7 (3)
O1—Cu1—N2—C15	22.8 (3)	Cu1—N1—C10—C11	20.0 (4)
N1—Cu1—N2—C15	−157.0 (3)	C15—N2—C11—C10	179.6 (3)
N1—Cu1—N2—C11	−27.3 (2)	Cu1—N2—C11—C10	44.6 (3)
Cu1—O1—C1—O2	−3.8 (4)	C15—N2—C11—C12	−54.0 (5)
Cu1—O1—C1—C2	174.7 (3)	Cu1—N2—C11—C12	171.0 (3)
Cu1—O3—C3—C8	13.7 (5)	N1—C10—C11—N2	−42.4 (4)
Cu1—O3—C3—C4	−167.5 (3)	N1—C10—C11—C12	−168.6 (3)
O3—C3—C4—C5	178.3 (4)	N2—C11—C12—C13	53.5 (5)
C8—C3—C4—C5	−2.8 (6)	C10—C11—C12—C13	176.8 (3)
C3—C4—C5—C6	1.6 (8)	C11—C12—C13—C14	−52.1 (5)
C4—C5—C6—C7	0.9 (8)	C12—C13—C14—C15	52.5 (5)
C5—C6—C7—C8	−2.1 (8)	C11—N2—C15—C14	52.9 (4)
C6—C7—C8—C3	0.8 (7)	Cu1—N2—C15—C14	−179.5 (2)
C6—C7—C8—C9	178.3 (4)	C13—C14—C15—N2	−53.4 (5)
O3—C3—C8—C7	−179.5 (3)		

### Hydrogen-bond geometry (Å, º)

**Table:**

*D*—H···*A*	*D*—H	H···*A*	*D*···*A*	*D*—H···*A*
O1*W*—H1*W*2···O2^i^	0.79 (6)	2.06 (6)	2.845 (5)	173 (6)
O1*W*—H1*W*1···O3^ii^	0.81 (8)	2.24 (9)	2.970 (6)	151 (8)
N2—H1*N*···O1*W*^iii^	1.00 (5)	2.09 (5)	3.047 (5)	159 (4)

Symmetry codes: (i) −*x*+2, −*y*+1, −*z*+1; (ii) *x*, *y*, *z*−1; (iii) −*x*+1, −*y*+1, −*z*+1.
